# HEXIM1-Tat chimera inhibits HIV-1 replication

**DOI:** 10.1371/journal.ppat.1007402

**Published:** 2018-11-05

**Authors:** Marie Leoz, Petra Kukanja, Zeping Luo, Fang Huang, Daniele C. Cary, B. Matija Peterlin, Koh Fujinaga

**Affiliations:** Department of Medicine, Microbiology and Immunology, UCSF, San Francisco, California, United States of America; Duke University Medical Center, UNITED STATES

## Abstract

Transcription of HIV provirus is a key step of the viral cycle, and depends on the recruitment of the cellular positive transcription elongation factor b (P-TEFb) to the HIV promoter. The viral transactivator Tat can displace P-TEFb from the 7SK small nuclear ribonucleoprotein, where it is bound and inactivated by HEXIM1, and bring it to TAR, which allows the stalled RNA polymerase II to transition to successful transcription elongation. In this study, we designed a chimeric inhibitor of HIV transcription by combining functional domains from HEXIM1 and Tat. The chimera (HT1) potently inhibited gene expression from the HIV promoter, by competing with Tat for TAR and P-TEFb binding, while keeping the latter inactive. HT1 inhibited spreading infection as well as viral reactivation in lymphocyte T cell line models of HIV latency, with little effect on cellular transcription and metabolism. This proof-of-concept study validates an innovative approach to interfering with HIV transcription via peptide mimicry and competition for RNA-protein interactions. HT1 represents a new candidate for HIV therapy, or HIV cure via the proposed block and lock strategy.

## Author summary

HIV remains a major health issue, with still no vaccine or cure available, and lifelong antiretroviral treatment required for the always-increasing number of people living with the virus. Combination antiretroviral therapy inhibits HIV replication, but the persistence of latently infected cells remains a challenge. In this study, we developed a new approach to inhibiting HIV transcription with a chimera derived from host and viral proteins involved in the regulation of HIV gene expression. We fused a domain from the viral transactivator Tat to two domains from the host cell transcription regulator HEXIM1. The chimera (HT1) binds to TAR, inhibits P-TEFb, and prevents Tat transactivation of the HIV promoter. Cellular genes are not impacted. When stably expressed by lymphocyte T cells, the chimera potently inhibits HIV replication and reactivation from latency, which makes it a promising candidate for therapy or cure by a block and lock approach.

## Introduction

Treatment with combination antiretroviral therapy (cART) leads to efficient suppression of HIV replication, but HIV persistence in latently infected cells remains an obstacle to cure [1]. Even under cART, residual HIV replication can arise and ultimately lead to the emergence of replicative resistance mutations and viral escape. Targeting diverse steps of the viral life cycle is the most efficient way to prevent viral escape. Currently, viral entry, reverse transcription, integration and maturation steps have been targeted by cART [2]. However, no efficient transcription inhibitor is clinically available, though multiple strategies–such as TAR decoys [3] or dominant-negative Tat [4]—have been explored to prevent expression of the integrated provirus.

Blocking transcription would not only add another therapeutic target, but also prevent sporadic reactivation of integrated HIV [5] that may contribute to HIV persistence, reservoir replenishment and chronic inflammation [6–8]. Suppressing residual HIV transcription is also the goal of the emerging block and lock HIV cure strategies [9–11], which aim at deepening HIV latency so that integrated proviruses remains permanently locked in the infected cells. Several latency promoting agents (LPAs) have been proposed, such as didehydro-cortistatin A [10], curaxin 100 [11], ruxolitinib and tofacitinib [12]. More studies are needed to determine whether a permanent state of latency can actually be reached after LPA treatment is interrupted. This would validate block and lock strategies as a path to a functional cure and/or a faster reservoir decay, a process that is probably delayed by residual replication and cell proliferation [13].

**Fig 1:**
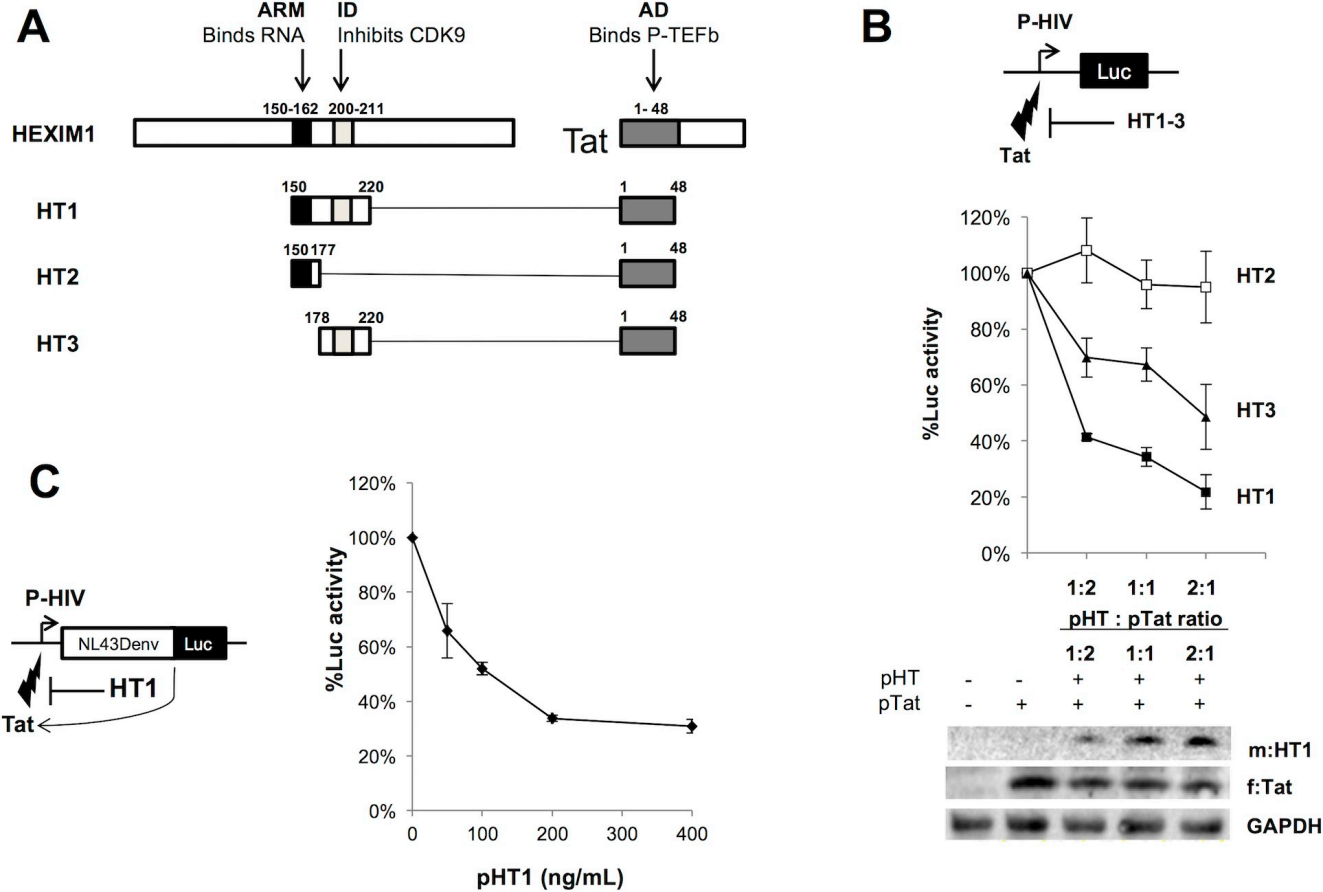
A HEXIM1-Tat fusion peptide inhibits gene expression from the HIV promoter. **A. Structure of the HEXIM1-Tat fusion peptides used in the study.** The functional domains used from HEXIM1 and Tat include a HEXIM1 Arginine Rich Motif (ARM, black box, residues 150–162) that binds RNA, a HEXIM1 inhibitory domain (ID, light grey box, residues 200–211) that inhibits CDK9 through a PYNT motif, and Tat transactivation domain (AD, dark grey box, residues 1–48) that binds to P-TEFb. The conceptual schemes are not drawn to scale. **B. Transient expression of HT1 inhibits Tat-induced LTR-driven Luc expression.** Upper panel: a schema depicts the reporter assay. A luciferase reporter gene (Luc) is under the control of the HIV promoter (P-HIV) and can be activated by ectopically expressed Tat. We use a Luc assay to titrate the inhibitory effect of HT1-3 on P-HIV transactivation by Tat. Middle panel: increasing amounts of m:HT1, m:HT2 or m:HT3 expressing plasmid (pHT) were co-transfected in 293T cells with a plasmid (pTat) expressing f:Tat and another (pLTR-Luc) expressing Luc under the control of the HIV promoter. Luc activity was plotted as % activity relative to control (empty vector used instead of pHT), depending on the transfected pHT : pTat ratio. Error bars in the graph represent standard deviation from triplicate experiments. Lower panel: expression levels of m:HT1 and f:Tat were confirmed by WB using anti-Myc and anti-Flag Abs. Housekeeping protein GAPDH was used as loading control. **C. Transient expression of HT1 inhibits Luc expression from HIV-1 NL43ΔenvLuc.** Left panel: a schema depicts the reporter assay. Luc is inserted in a replication-defective HIV-1 molecular clone (pNL43**Δ**envLuc) in which Luc and Tat are under the control of the HIV promoter (P-HIV). We use a Luc assay to titrate the inhibitory effect of HT1 on P-HIV transactivation by Tat. Right panel: increasing amounts of pHT1 were co-transfected in 293T cells with pNL43**Δ**envLuc. Luc activity was plotted as % activity relative to control (empty vector used instead of pHT), depending on the transfected amounts of pHT1. Error bars in the graph represent standard deviation from triplicate experiments.

HIV expression is dependent on the viral transactivator Tat, which brings the cellular positive transcription elongation factor B (P-TEFb) to the HIV promoter [14, 15]. P-TEFb is comprised of cyclin T1 (CycT1) and cyclin-dependent kinase 9 (CDK9) [16, 17], and is required for transcription elongation, both for HIV and host gene expression. In cells, most of P-TEFb is sequestered in the 7SK small nuclear ribonucleoprotein complex (7SK snRNP), which includes a non-coding 7SK snRNA and proteins HEXIM1, LARP7 and MePCE [18, 19]. In 7SK snRNP, the transcriptional regulator HEXIM1 binds to the 7SK snRNA through a RNA-binding arginine rich motif (ARM, residues 150–162, see Fig 1A), and to P-TEFb through its CycT1 binding domain (TBD, residues 250–359 [20, 21] and its central inhibitory domain (ID, residues 200–211, Fig 1A). This ID includes a PYNT motif (^202^Pro-^203^Tyr-^204^Asn-^205^Thr), which masks CDK9’s substrate-binding site and is critical for its inactivation [22, 23]. Importantly, HEXIM1’s TBD acts synergistically with ID on CDK9 inhibition [24].

Without recruitment of active P-TEFb to the HIV promoter, RNA polymerase II (RNAPII) is stalled after having only transcribed the short transactivation response element (TAR) RNA, located at the 5’ end of all viral transcripts [15, 25]. The Tat activation domain (AD, residues 1–48, see Fig 1A) binds CycT1, while its central ARM binds to 7SK RNA, thus displacing P-TEFb from the 7SK snRNP and releasing it from HEXIM1 inhibition [26]. Tat and CycT1 also form a cooperative binding surface for TAR, where the central ARM region of Tat (residues 51–57) binds to the bulge region of TAR and the Tat-TAR recognition motif of CycT1 binds to the central loop of TAR [27]. These interactions allow P-TEFb to be recruited to the RNAPII early elongation complex that is stalled at the HIV transcription start site. There, CDK9 phosphorylates transcriptional inhibitory complexes NELF and DSIF as well as RNAPII C-terminal domain (CTD), resulting in stimulation of transcription elongation [5, 28].

Interestingly, Tat/TAR/P-TEFb interaction structurally mimics that of HEXIM1/7SK/P-TEFb, and the amino-acid sequences of Tat and HEXIM1 ARMs are almost identical [22]. Since the former is a strong transcription activator for HIV, while the latter is a potent inhibitor of P-TEFb, we sought to create a HIV-specific transcription inhibitor by taking advantage of the structure similarities of these two complexes. We designed chimeras that derive from critical functional domains of Tat and HEXIM1, by combining the P-TEFb binding N-terminal domain of Tat to the acidic and/or central basic domains of HEXIM1 that respectively inhibit P-TEFb and bind RNA. A small HEXIM1-Tat chimera, HT1, inhibited HIV transcription by preventing the recruitment of active P-TEFb to TAR, with only little off-target effects on cellular genes. This proof of concept study demonstrates the feasibility of designing highly specific transcriptional inhibitor chimeras.

## Results

### A HEXIM1/Tat chimera inhibits transcription from the HIV promoter

We screened a collection of chimeras (Fig 1A and S1 Fig) derived from the ARM and/or ID of HEXIM1 fused to the AD of Tat (Tat1-48). These chimeras include: HT1 with both HEXIM1 domains of interest (Hex150-220), HT2 with only HEXIM1 ARM (Hex150-177) and HT3 with only HEXIM1 ID (Hex178-220).

Luciferase (Luc) reporter assays were performed to titrate the potency of each chimera to inhibit Tat-dependent gene expression from the HIV promoter (Fig 1B, upper schema). Effector plasmids included pHT1-3 and pTat, which respectively expressed a Myc-epitope tag chimera (m:HT1-3) and a Flag-epitope tagged Tat (f:Tat). pHT1, pHT2 or pHT3 was transiently co-transfected in 293T cells with pTat and a Luc reporter gene under the control of the HIV promoter (P-HIV, expressed from the plasmid pLTR-Luc) (Fig 1B, upper panel). HT2, which does not include HEXIM1 ID, failed to inhibit Luc expression from the HIV promoter, even when the ratio of pHT2 to pTat was 2:1 (Fig 1B, middle panel). HT3, which included the ID, induced up to a 2-fold decrease in Luc expression from the HIV promoter at the 2:1 ratio (Fig 1B, middle panel). Finally, HT1 lead to a 4-fold decrease in Luc expression at a 2:1 ratio (Fig 1B, middle panel). The stronger inhibitory effect by HT1 when compared to that by HT3 suggested that HEXIM1 ARM also contributes to the inhibition of Tat-induced Luc expression from the HIV promoter. Importantly, mutating the PYNT motif, which is critical for HEXIM1's CDK9 inhibition, abolished the inhibitory effect of HT1 (HT1.PNND in S1 and S2 Figs, bar 3), suggesting that HT1's inhibitory effect on HIV transcription is mediated by CDK9 inhibition. Adding different lengths of flexible peptide linkers (GGGGS) slightly, but insignificantly improved the inhibitory effects of HT1 (S1 and S2A Figs, bars 4 and 5). Also, reversing the order of peptide motif (1TH in S1 Fig) also abolished HT1's inhibitory effect (S2A Fig, bar 7), indicating that precise spatial arrangement of these motifs is required. Similar results were obtained when co-transfecting pHT1-3 and pTat in NIH1 cells, which stably carry an LTR-Luc reporter gene [29] (S2B Fig). Thus, we selected HT1 for further investigation.

Levels of protein expression from pHT1 and pTat in 293T cells were confirmed by western blotting (WB, Fig 1B, lower panel) and suggested that the expression of Tat may be slightly reduced upon co-transfection of pHT1. To rule out any bias in the inhibitory titration of HT1, we thus used pNL43**Δ**envLuc, a defective HIV molecular clone from which Tat is expressed from the HIV promoter and not from a separate plasmid. In this model, transient expression of HT1 led to a 3-fold decrease in Luc expression (Fig 1C). Taken together, these results suggest that HT1 potently inhibits gene expression from the HIV promoter.

### HT1 prevents Tat from bringing active P-TEFb to TAR

**Fig 2:**
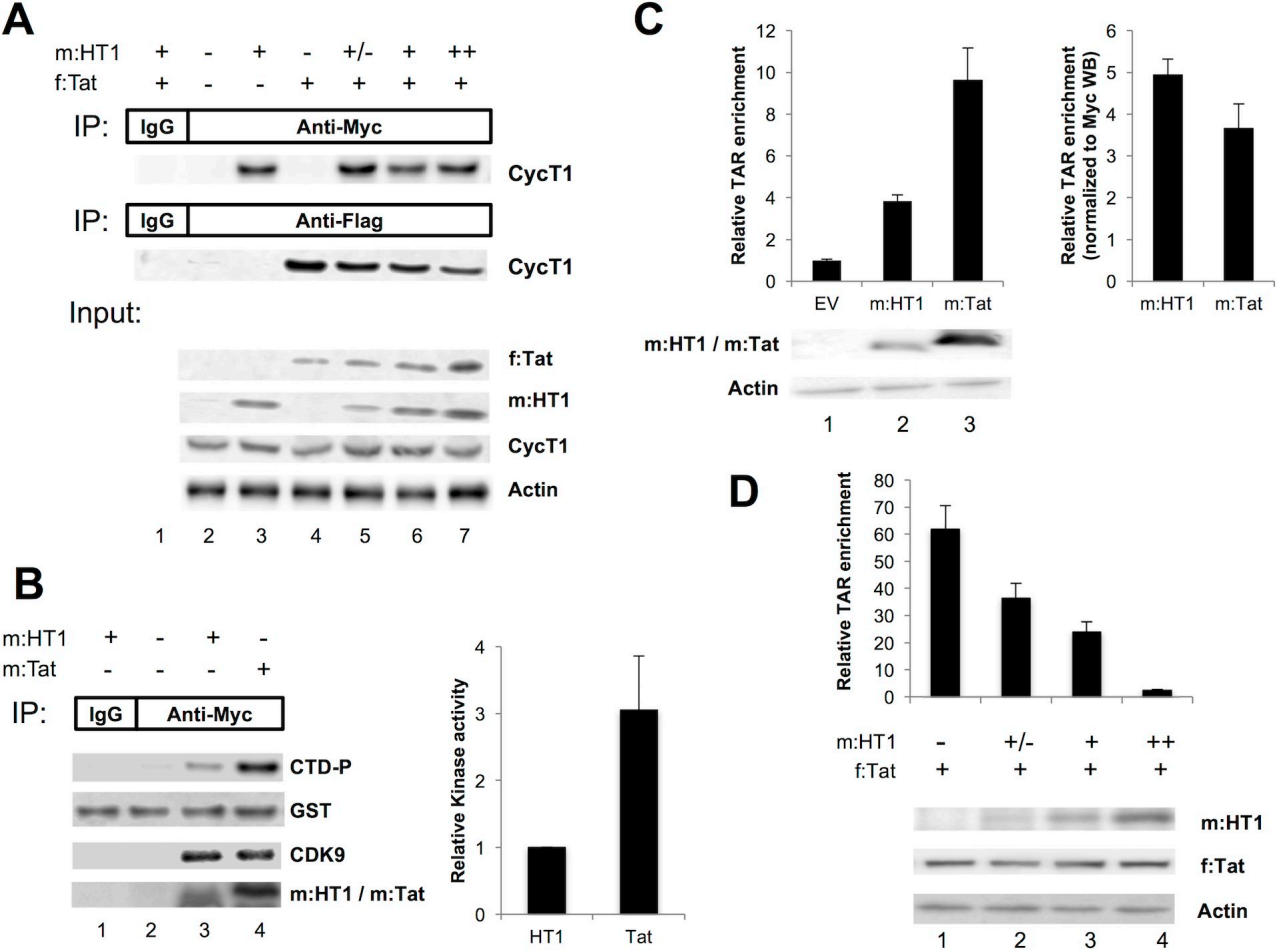
HT1 prevents Tat from bringing active P-TEFb to TAR. **A. HT1 binds to P-TEFb and competes with Tat for P-TEFb binding.** m:HT1 and/or f:Tat were transiently expressed in 293T cells. Cell lysates were used for immunoprecipitation (IP) using anti-Myc Ab (upper panel, lanes 2–7), anti-Flag Ab (middle panel, lanes 2–7), or control IgG (upper and middle panels, lane 1). Input lysates (lower panel) and immunoprecipitates were submitted to SDS-PAGE and WB using anti-CycT1, anti-Myc, anti-Flag and anti-actin Abs. **B. HT1 inhibits the kinase activity of P-TEFb subunit CDK9.** m:HT1 and/or f:Tat were transiently expressed in 293T cells. Cell lysates were used for IP using anti-Myc Ab (lanes 2–4), or control IgG (lane 1). Immunoprecipitates were incubated with ATP and recombinant GST-CTD proteins for *in vitro* kinase assay. Total GST-CTD was detected using anti-GST Ab and phosphorylated GST-CTD (CTD-P) was detected using anti-Ser2P Ab. m:HT1 and m:Tat were detected using anti-Myc Ab. Six replicate experiments were performed, and mean relative kinase activities are shown in the bar graph to the right. **C. HT1 binds to TAR.** m:HT1 or m:Tat (or empty vector, EV, as a control) was transiently co-expressed with TAR RNA-expressing pU16TAR in 293T cells. Cell lysates were used for IP using anti-Myc Ab or control IgG. RNA was purified from the immunoprecipitates and submitted to RT-qPCR using TAR-specific primers (upper left panel). Relative TAR enrichment was calculated as qPCR count using anti-Myc Ab minus using IgG, and normalized to EV. Error bars represent standard deviation from triplicate qPCR assays. Input lysates were submitted to SDS-PAGE and WB using anti-Myc and anti-Actin Abs (lower left panel). The amounts and standard deviations of immunoprecipitated TAR RNA (from the upper left panel) were normalized to the respective amount of m:HT1 or m:Tat detected in the input lysate (right panel). **D. HT1 competes with Tat for TAR binding.** m:HT1 and/or f:Tat were transiently co-expressed with TAR RNA-expressing pU16TAR in 293T cells. Cell lysates were used for IP using anti-Myc Ab or control IgG. RNA was purified from the immunoprecipitates and submitted to RT-qPCR using TAR-specific primers (upper panel). Relative TAR enrichment was calculated as qPCR count using anti-Myc Ab minus using IgG, and normalized to EV. Error bars represent standard deviation from triplicate qPCR assays. Input lysates were submitted to SDS-PAGE and WB using anti-Myc, anti-Flag and anti-Actin Abs (lower panel).

We next investigated the mechanisms by which HT1 can prevent HIV gene expression. Since both Tat AD and HEXIM1 ID interact with P-TEFb, we performed a series of co-immunoprecipitations to determine whether HT1 interacted with P-TEFb and competed with Tat for P-TEFb binding. m:HT1 and f:Tat were transiently expressed in 293T cells, and both bound to CycT1 as demonstrated using anti-Myc or anti-Flag antibodies (Abs) for co-immunoprecipitation (Fig 2A, lane 3 in upper panel and 4 in middle panel, respectively). Moreover, when co-expressing a fixed amount of f:Tat and increasing amounts of m:HT1 (Fig 2A, lanes 5–7, lower panel), the amounts of CycT1 co-immunoprecipitating with f:Tat decreased (Fig 2A, lanes 5–7, middle panel), suggesting a competition between HT1 and Tat for P-TEFb binding.

Next we investigated whether HT1, which contains HEXIM1 ID, inhibited the kinase activity of CDK9. m:HT1 was transiently expressed in 293T cells and immunoprecipitated using anti-Myc Ab. Co-immunoprecipitated P-TEFb was subjected to an *in vitro* kinase assay with ATP and recombinant GST-CTD proteins as a substrate. Similarly, m:Tat was expressed to co-immunoprecipitate P-TEFb as a positive control. Immunoprecipitated CDK9, and phosphorylated GST-CTD (CTD-P) were detected by WB using anti-CDK9 and anti-Ser2P Abs, respectively (Fig 2B). A larger amount of CDK9 was co-immunoprecipitated by HT1 than by Tat, while more CTD-P was detected with Tat than with HT1 (Fig 2B, left panel, lanes 3 and 4). Relative kinase activity associated with HT1 and Tat was calculated by CTD-P band intensity normalized with CDK9, which revealed that the kinase activity of P-TEFb was decreased 3.1 fold when bound to HT1, compared to control (Fig 2B, right panel). This suggests that once bound to P-TEFb, HT1 can inhibit the kinase activity of CDK9. Consistently, addition of another non-inhibitory P-TEFb-binding motif (PID) from Brd4 to HT1 decreased the ability to inhibit HIV transcription (PID.HT1 in S1 and S2 Figs, bar 6).

Finally, since HEXIM1 ARM resembles the TAR-binding domain from Tat, we investigated whether HT1 could bind to HIV TAR. m:HT1 or m:Tat was co-expressed in 293T cells with TAR RNA, expressed under RNA Polymerase III dependent H1 promoter [3], and immunoprecipitated using anti-Myc Ab or IgG as a control. TAR RNAs co-immunoprecipitated with HT1 or Tat were quantified by RT-qPCR analysis. Both HT1 and Tat immunoprecipitated TAR (Fig 2C, upper left panel, lanes 2 and 3), though m:Tat immunoprecipitated 2.54 fold more TAR than m:HT1. Since the level of m:Tat expressed in cell lysates was higher than that of m:HT1 (Fig 2C, lower left panel, lanes 2 and 3), we normalized the amount of immunoprecipitated TAR by the protein levels of m:HT1 and m:Tat, which suggested that similar amounts of m:HT1 bound at least as much TAR as m:Tat (Fig 2C, right panel). m:HT3, which lacked the ARM domain from HEXIM1 (Fig 1A), failed to bind TAR (S3A Fig). To test whether HT1 affects Tat-TAR interactions, increasing amounts of m:HT1 were also co-expressed with a fixed amount of f:Tat and TAR (Fig 2D). RNA immunoprecipitation assays were then performed using anti-Flag Ab followed by TAR RT-qPCR, and indicated that the amounts of TAR RNA co-immunoprecipitated with f:Tat decreased progressively when expression of m:HT1 increased (Fig 2D, upper panel). WB confirmed that the amounts of HT1 used for this assay did not impact on f:Tat expression (Fig 2C, lower panel), suggesting that the decrease in co-immunoprecipitated TAR was due to HT1 competing with Tat for TAR binding. As expected, HT1 also bound to endogenous 7SK snRNA (S3B Fig).

These results confirm that HT1 competes with Tat for P-TEFb binding and keeps its kinase subunit CDK9 inactive, which reduces the amount of P-TEFb that is available for Tat to bring to TAR. Moreover, HT1 also binds to TAR and prevents Tat from binding it, consistent with the observation in Fig 1B of a more potent inhibition by HT1 than by HT3. Two mechanisms are thus combined that prevent Tat from bringing active P-TEFb to TAR for successful HIV transcription elongation.

### Expression of HT1 does not impair host cell gene expression and growth

**Fig 3:**
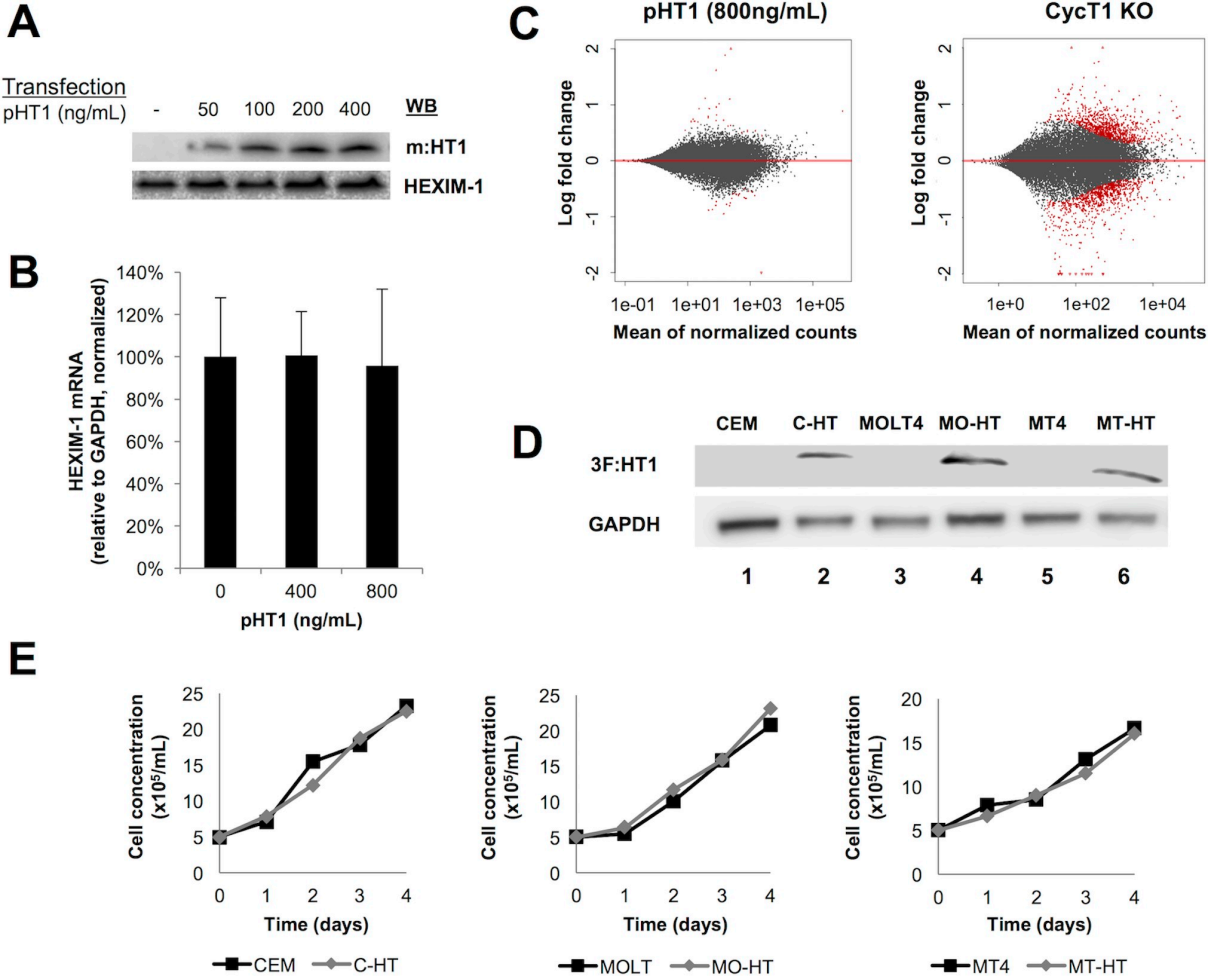
Expression of HT1 doesn’t impair cell gene expression and growth. **A. Transient expression of HT1 doesn’t impair P-TEFb dependent expression of endogenous HEXIM1 protein in 293T cells.** Increasing amounts of m:HT1 were transiently expressed in 293T cells. Cell lysates were submitted to SDS-PAGE and WB using anti-Myc and anti-HEXIM1 Abs. **B. Transient expression of HT1 doesn’t impair P-TEFb dependent transcription of endogenous hexim1 gene in 293T cells.** Increasing amounts of m:HT1 were transiently expressed in 293T cells. Cellular RNA was purified and submitted to RT-qPCR using HEXIM1 and GAPDH specific primer pairs. The amount of HEXIM1 mRNA was normalized to that of GAPDH. Error bars represent standard deviation from triplicate experiments. **C. Transient expression of HT1 doesn’t impair global gene expression in 293T cells.** HT1 was transiently expressed in 293T cells. Cellular RNA was purified from these cells as well as from control 293T cells and CycT1 KO 293T cells, and submitted to mRNAseq. The upper panel shows Log2 fold change difference in gene expression between HT1-expressing cells and control cells, ranked by mean of normalized cell counts. Lower panel shows the same analysis for CycT1 KO cells relative to control. Red dots correspond to genes that are differentially expressed in HT1-expressing cells or in CycT1 KO cells compared to control (padj<0,1). Each condition was tested in triplicate experiments. **D. Stable expression of HT1 in CEM, MOLT4 and MT4 cells** 3f:HT1 was stably expressed in CEM, MOLT4, or MT4 cells (C-HT, MO-HT, MT-HT, respectively). Cell lysates were submitted to SDS-PAGE and WB using anti-Flag and anti-GAPDH Abs. **E. Stable expression of HT1 doesn’t impair cell growth of C-HT, MO-HT and MT-HT cells.** 100,000 cells were seeded in 2 mL RPMI with 10% FBS, and with 1 μg/mL puromycin for HT-expressing cells. Total viable cell count was performed at days 0–4 to assess cell growth. Error bars represent standard deviation from triplicate experiments.

Since P-TEFb is a major transcription factor and regulates the expression of many cellular genes, we next assessed whether HT1 could impair host cell gene expression through inactivation of the kinase activity of CDK9. We first investigated how HT1 impacted the mRNA and protein expression levels of HEXIM1, a bona fide target of P-TEFb [30]. Increasing amounts of m:HT1 were transiently expressed in 293T cells and did not change the expression level of HEXIM1 protein (Fig 3A) and mRNA (Fig 3B).

The specificity of HT1 was further investigated by mRNA-seq analysis in 293T cells. Only 48 genes were differentially expressed upon ectopic expression of HT1 (26 up-regulated and 22 down-regulated with padj < 0.1, shown in red in Fig 3C, upper panel), while knocking out CycT1 as a control impacted 1673 genes (shown in red in Fig 3C, lower panel). A third of the genes impacted by HT1 expression corresponded to up-regulated non-coding RNAs, including 7SK (fold-change = 1.1, padj = 9.6E-06), an effect that may be due to a stabilization of these RNAs.

Finally, the impact of HT1 on cell growth was assessed using three T cell lines (CEM, MOLT4, and MT4) infected by a lentivirus expressing a triple Flag-epitope tagged HT1 (3f:HT1). Polyclonal population of HT1-expressing cells (C-HT, MO-HT, and MT-HT, respectively) was selected by puromycin and confirmed by WB (Fig 3D). Total viable cell count over time showed no difference in cellular growth rate between HT1-expressing and control cells (Fig 3E). This confirmed that HT1 was specific to HIV inhibition, and that the few off-target effects had little impact on the metabolism of the cells.

#### Stable expression of HT1 inhibits HIV reactivation and replication

**Fig 4:**
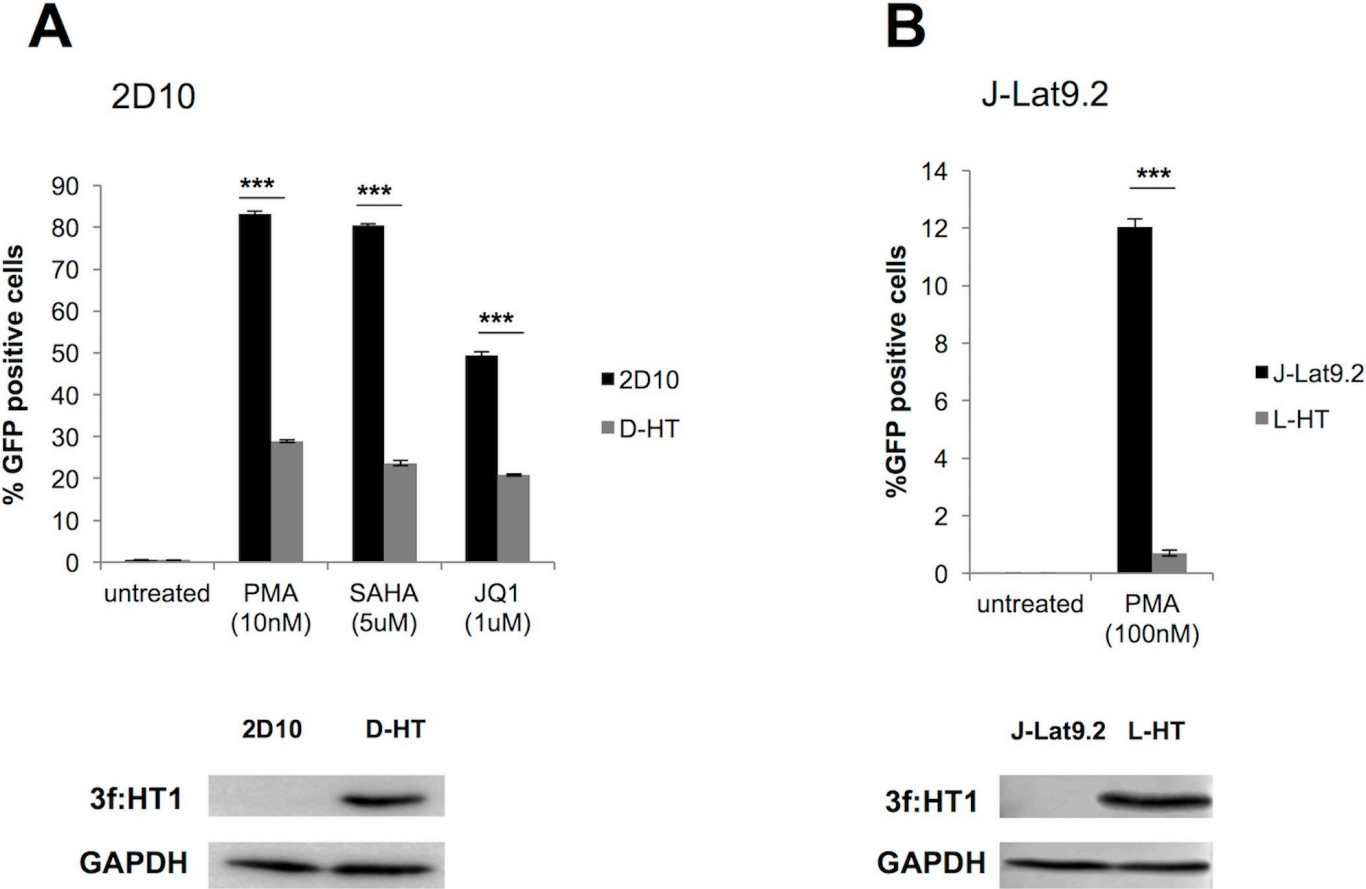
Stable expression of HT1 inhibits HIV reactivation. **A. Stable expression of HT1 inhibits HIV reactivation from 2D10 cells.** 3f:HT1 was stably expressed in 2D10 cells (D-HT cells). Cell lysates were submitted to SDS-PAGE and WB using anti-Flag and anti-GAPDH Abs (lower panel). FACS analysis of GFP positive cells showed that HIV reactivation by PMA, SAHA or JQ1 was significantly impaired in D-HT cells when compared to 2D10 cells, as determined by a student t-test. *** represent differences with p < E-03 (p = 4.75E-05, p = 4.35E-05 and p = 7.62E-05 respectively). Error bars represent standard deviation from triplicate FACS analysis. **B. Stable expression of HT1 inhibits HIV reactivation from J-Lat 9.2 cells**. 3f:HT1 was stably expressed in J-Lat 9.2 cells (L-HT cells). Cell lysates were submitted to SDS-PAGE and WB using anti-Flag and anti-GAPDH Abs (lower panel). FACS analysis of GFP positive cells showed that HIV reactivation by PMA was significantly impaired in L-HT cells when compared to J-Lat 9.2 cells, as determined by a student t-test. *** represent differences with p < E-03 (p = 5.62E-05). Error bars represent standard deviation from triplicate FACS analysis.

To test the effect of HT1 on HIV-1 in T-lymphocyte derived cells, 3f:HT1 was stably expressed in HIV latent infection models 2D10 [31] and J-Lat 9.2 [32] cells (D-HT and L-HT cells respectively, Fig 4A and Fig 4B, right panels), which carry replication-defective, GFP-flagged HIV proviruses. In both 2D10 and J-Lat 9.2, the basal HIV transcription level is undetectable, as measured through GFP expression. Compounds such as the PKC agonist PMA, the histone deacetylase inhibitor SAHA or the BET bromodomain inhibitor JQ1 [14] reactivate HIV transcription and increase GFP-positive cells detected by FACS analysis. 2D10 cells are more sensitive to HIV reactivation than J-Lat 9.2 cells, though they harbor a mutation in the N-terminal sequence of Tat (H13L [31]). 2D10 and D-HT cells were incubated for 24 hrs with PMA (10 nM), SAHA (5 μM) or JQ1(1 μM) and GFP positive cells were detected by FACS analysis. PMA, SAHA and JQ1 reactivated HIV from 65%, 71% and 58% less D-HT cells than from control 2D10 cells, respectively (Fig 4A, left panel). Due to the limited HIV reactivation by SAHA and JQ1 in J-Lat 9.2 cells, we only treated them with PMA (100 nM), which induced a 12% increase in GFP expression (Fig 4B, left panel, black bar). Stable expression of HT1 resulted in a 94% decrease of GFP expression in L-HT compared to J-Lat 9.2 (Fig 4B, left panel, grey bar). Taken together, these results show that stable expression of HT1 significantly impairs HIV reactivation in latently infected T cells, consistent with previous observation that HT1 can inhibit HIV gene expression.

**Fig 5:**
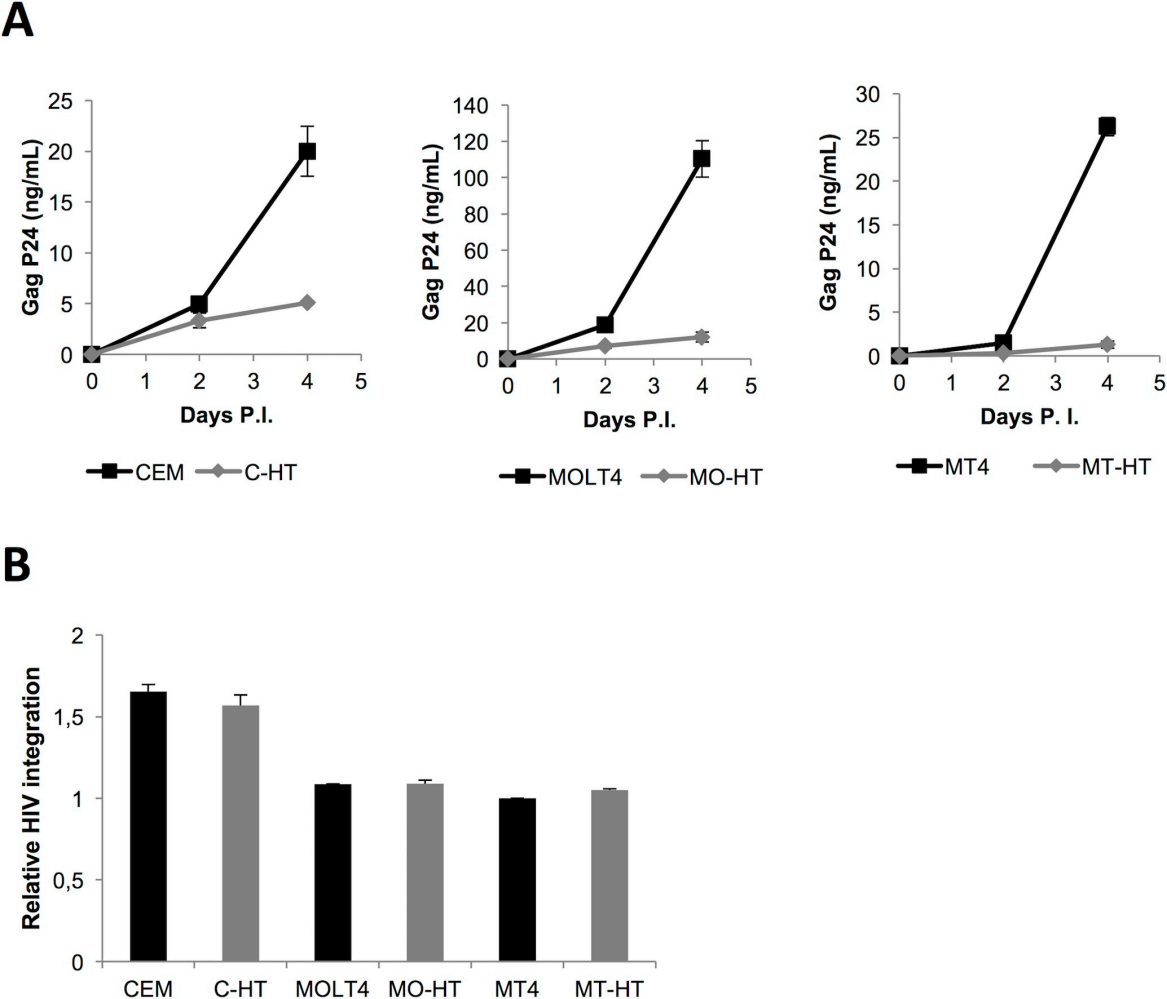
Stable expression of HT1 inhibits HIV replication. **A. Stable expression of HT1 inhibits HIV-1 replication in C-HT, MO-HT, and MT-HT cells.** 1E+06 cells (HT1-expressing CEM-HT, MOLT4-HT, MT4-HT and respective controls CEM, MOLT4 and MT4 cells) were challenged by HIV-1 NL43 infection. Virus production was assessed by Gag p24 ELISA in supernatants when cells were passaged at days 0, 2 and 4, and plotted. Error bars represent standard deviation from triplicate experiments. **B. Stable expression of HT1 doesn’t inhibit early steps of HIV-1 infection in C-HT, MO-HT, and MT-HT cells.** Single-round infection assays were performed in the same conditions as in Fig 5A above, but using replication-defective HXB2 Env-pseudotyped HIV-1 NL43ΔenvLuc. The relative amount of HIV DNA integrated in the cells’ genomic DNA was assessed after 24h by qPCR. Error bars represent standard deviation from triplicate experiments.

To investigate whether HT1 also inhibits HIV in a spreading infection, we infected the C-HT, MO-HT, and MT-HT cells with wild type HIV-1 NL43 virus, and collected the supernatants on 0, 2 and 4 days post infection (dpi) to assess viral production by Gag p24 ELISA (Fig 5A). On 2 dpi, virus production was detected from CEM, MOLT4 and MT4 cells and reached around 20 (CEM, MT4) to 110 (MOLT4) ng/mL p24 concentration in the supernatant (Fig 5A, black lines) on 4 dpi. There was only 1.3 ng/mL, 5.1 and 12 ng/mL p24 concentration in the MT-HT, C-HT and MO-HT supernatants on 4 dpi (Fig 5A, grey lines), indicating that stable expression of HT1 reduced HIV replication by 75 to 95% in these cells. Single-round infection assays using HIV Env-pseudotyped replication-defective HIV, followed by measurement of proviral DNA by qPCR, indicated that early steps of HIV infection were not affected by HT1 expression (Fig 5B). Together with the HIV reactivation assays, these results confirm that HT1 is a potent inhibitor of HIV gene expression and replication.

## Discussion

In this study, we developed a new approach to block HIV transcription. We designed a chimera (HT1) containing the RNA-binding (ARM) and CDK9-inhibitory (ID) domains from the transcription regulator HEXIM1, and the P-TEFb-binding domain from the viral transactivator Tat (AD). Consistent with the respective properties of these domains in the context of their original proteins, HT1 competed with Tat for P-TEFb- and TAR-binding, and kept P-TEFb inactive. As a consequence, HT1 prevented Tat from bringing active P-TEFb to TAR for successful transcription elongation, as confirmed by the potent inhibition of HIV gene expression and replication. The use of a Tat-derived domain also conferred HT1 a high level of specificity, with little impact on host gene transcription and metabolism.

In this proof-of-concept study, diverse T-lymphocyte derived cell line models of HIV infection, including latent infection models, were preferred over primary CD4+ T cells in which production and selection of HT1-expressing cells would be challenging. We used plasmid or lentiviral delivery of HT1, which would require major technical adjustments to translate to a primary cell model. The very low amounts of transcription factors in these cells, including P-TEFb, would impair HT1 expression and inhibitory effect–even though the titrations in Fig 1B and Fig 1C suggest that effective HIV inhibition may be reached even at low HT1 expression levels. Another strategy would be to transduce the peptide into primary cells, which will also require optimization due to the oxidation-prone Cystein-rich Tat domain included in the chimera [33]. Although beyond the scope of the present study, determining the optimal conditions for successful delivery will thus be needed for any clinical application of HT1.

Despite this limitation, we validated a new strategic approach in HIV therapy: we used the fundamental knowledge in the structure and function of proteins involved in Tat-dependent HIV transcription for a logical design of an inhibitory peptide. ID of HEXIM1 contains the PYNT motif which is critical for inhibition of CDK9 kinase activity [20]. However, the C-terminal CycT1-binding domain (TBD) also contributes to the CDK9-inhibition by HEXIM1 [24]. Since the AD of Tat has a higher affinity to CycT1 than HEXIM1 [26], replacing HEXIM1's TBD with Tat AD was expected to make HT1 able to compete with HEXIM1 for P-TEFb binding. Adding HEXIM1 ARM to HT1 also made it able to compete with Tat ARM for TAR binding. The three domains were thus needed for HT1 to efficiently compete with HEXIM1 and Tat, so that it potently inhibits P-TEFb-induced HIV transcription elongation. In addition, spatial arrangement of these domains was critical for the inhibitory activity (S2 Fig), suggesting requirement for a precise placement of ID between the RNA- and P-TEFb-binding domains, as is naturally the case in HEXIM1. However, and as opposed as HEXIM1, HT1 did not need the C-terminal coiled-coil domain that is required for HEXIM1 dimerization and inhibitory activity [34]. Indeed, we showed that HT1 successfully competed with Tat in both Tat/TAR/P-TEFb and HEXIM1/7SK/P-TEFb complexes, and kept bound P-TEFb inactive. HT1 was especially efficient in competing for TAR binding (Fig 2D), which may be due to higher affinity for RNA through a longer and more basic ARM than Tat’s. Since an efficient Tat/TAR/P-TEFb interaction involves the Tat-TAR recognition motif of CycT1, HT1 competing for P-TEFb binding may also contribute to the competition for TAR-binding. Our study hence demonstrated that HT1 properties precisely match assumptions derived from each piece of biochemical data on Tat/TAR/P-TEFb and HEXIM1/7SK/P-TEFb interactions. Precise design can therefore make peptide therapy more specific, and thus better tolerated than the small molecules most often used in cART, as confirmed by the only minor off-target effects of HT1 with no impact on cell growth. Reactivation assays from two distinct HIV latency models showed that HT1 efficiently inhibited HIV reactivation by a broad range of latency reversal agents. This efficiency was further confirmed in spreading HIV infection assays, where HT1 not only competed with Tat, but prevented Tat to be expressed from the first round of transcription of newly integrated proviruses. This positive feedback loop of inhibition resulted in efficient inhibition of HIV replication after multiple rounds of infection.

Multiple therapeutic applications are possible for HT1, from prevention to therapy and cure. HT1 domains were derived from the human protein HEXIM1 and from HIV-1 Tat, neither of which are immunogenic, suggesting that clinical use of the chimera would be well tolerated. A key focus should be on investigating feasible cell delivery and route of administration. In an era of multiple and potent cART options, acceptability of a potentially injection-based treatment would mostly depend on the frequency and duration of administration, and may especially be fit for HIV cure application as opposed to long-term use as cART. Diverse options are now at hand in the fast evolving field of peptide therapeutics [35], including injection or alternative delivery routes such as oral or transdermal [36]. Gene therapy should also be considered, since we showed a positive inhibitory feedback loop in cells that stably expressed HT1 prior to HIV infection. In this model, HIV was virtually put into direct latency in newly infected cells, which in combination with blocking reactivation from pre-existing latently infected cells, could contribute to achieving a functional cure.

Finally, the design of such chimeras can be finely tuned to block other transcription factors that depend on P-TEFb. The CDK9-inhibiting module from HEXIM1 could indeed be combined with functional domains from transactivator targets other than Tat, allowing this strategy to be applied to other pathologies, including inflammation or cancer. This study thus paves the way to multiple applications of transcription-targeted inhibition peptide therapy.

## Methods

### Plasmids

Myc-epitope tagged HT1-3 (m:HT1-3) and Tat (m:Tat), and Flag-epitope tagged Tat (f:Tat) were inserted in the mammalian expression vector pEF-Bos [37], under the control of the EF1alpha promoter (plasmids referred to as pHT1-3 or pTat). For stable expression of HT1, triple Flag-epitope tagged HT1 (3f:HT1) was cloned in a 3^rd^ generation lentivirus gene expression vector (VectorBuilder). In this vector (pLHT1), HT1 and BFP were cloned under the control of the EF1a promoter, and a puromycin-resistance gene was also cloned under the control of mPGK promoter.

### Cells

Human Embryonic Kidney (HEK) 293T (ATCC) or 293T CycT1 knockout (KO) cells were grown in DMEM containing 10% Fetal Bovine Serum (FBS), at 37°C and 5% CO2. 293T CycT1 KO cells were created using the CRISPR/Cas9 system. All-in-one Cas9/sgRNA plasmid pSpCas9 BB-2A-Puro encoding sgRNA specific for CycT1 (sgRNA sequence: AAGCAGATTGGCCGCCTGC) (GenScript) was transfected into 293T cells using Lipofectamine 2000 (Invitrogen). After 48 hrs, untransfected cells were selected against by puromycin treatment for another 72 hrs. After puromycin selection, cells were cultured in normal media, and further cloned by limiting dilution. CycT1 KO clones were selected by analyzing CycT1 protein expression by WB using at least two different anti-CycT1 Abs (SCBT SC10750 and SC271348). Genomic CycT1 sequences were also analyzed to confirm that both alleles contained mutations that cause truncation of CycT1 protein at the N-terminus.

Human T-lymphocyte cell line CEM, MOLT4, MT4 cells (ATCC), and HIV-latently infected Jurkat cell clones J-Lat 9.2 [32] (given by Dr. Eric Verdin) or 2D10 [31] (given by Dr. Jonathan Karn) cells were grown in RPMI containing 10% FBS, at 37°C and 5% CO2. J-HT, L-HT and D-HT cells were produced by respectively infecting Jurkat, J-Lat 9.2 or 2D10 cells with a lentivirus (LHT1) produced by co-transfecting pLHT1 (see above), pMD2.G and psPAX2 (Addgene ID 12259 and 12260, respectively) in 293T cells using Polyplus transfection kit (Jetprime). 48 hrs after transfection, the supernatant from 293T cell culture was filtered, ultracentrifugated, and used for infection of CEM, MOLT4, MT4, J-Lat 9.2 or 2D10 cells (C-HT, MO-HT, MT-HT, L-HT and D-HT, respectively) in the presence of 2 μg/mL polybrene. C-HT, MO-HT, MT-HT, L-HT and D-HT cells were cultured in RPMI containing 10% FBS for 48 hrs, then puromycin (1 μg/mL) was added to the medium for antibiotic selection. Integration and stable expression of pLHT1 in C-HT, MO-HT, MT-HT, L-HT and D-HT cells was confirmed by BFP expression using FACS, and by WB using an anti-Flag Ab, as described below. Polyclonal populations of HT1-expressing cells were used throughout the study.

### Luciferase assays

pHT1-3 and/or pTat and/or empty pEF.Bos vector (see above) and a Luciferase reporter construct (whether pLTR-Luc or pNL43ΔenvLuc, both described in [38]) were transfected using Lipofectamine 2000 (Invitrogen) in 2.0E+05 293T cells. In Fig 1B transfection amounts were 0, 50, 100 or 200 ng/mL pHT1-3, 100 ng/mL pTat and 50 ng/mL pLTR-Luc, qsp 500 ng/mL using empty vector pEF-Bos. In Fig 1C transfection amounts were 0, 50, 100, 200 or 400 ng/mL pHT1 and 50 ng/mL pNL43ΔenvLuc, qsp 500 ng/mL using empty vector pEF-Bos. After 48 hrs, the cells were washed using PBS, lysed with Passive Lysis Buffer (Promega), and analyzed for Luciferase expression using D-Luciferin (BD Monolight) on an EG&G Berthold LB 96V microplate luminometer.

### Western blot

For detection of transiently expressed proteins, a total of 2.5 μg DNA, including pHT1-3 and/or pTat and/or empty pEF.Bos vector (see above), was transfected using Lipofectamine 3000 (Invitrogen) in 8.0E+05 293T cells. After 48 hrs, the cells were washed using PBS, and lysed using RIPA buffer containing 150 mM KCl and a protease inhibitor mixture (Thermo Fisher Scientific). Lysates were incubated for 10 min at 95°C in 2X Laemmli buffer (BioRad) supplemented with 5% DTT, and used for SDS-PAGE analysis in a 15% resolving gel. The proteins were then transferred to a PVDF membrane (BioRad), which was blocked in 5% milk in TBS and probed with specific primary Abs, which include anti-Myc (ab32 from mouse and ab9106 from rabbit, Abcam), anti-Flag (F7425 from mouse and F3165 from rabbit, Sigma-Aldrich), anti-GAPDH (GA1R, MA5-15738, Invitrogen), anti-actin (ab8227, Abcam), anti-CycT1 (SC-10570, SCBT), anti-HEXIM1 (25388, Abcam), anti-CDK9 (SC-484, SCBT), anti-phospho CTD (Ser 2) (ab5095, Abcam). Membranes were washed five times and incubated with peroxidase-conjugated secondary Abs, which include ECL mouse IgG HRP-linked whole Ab (NA9310, GE Healthcare) and ECL rabbit IgG HRP-linked whole Ab (NA9340, GE Healthcare). After five washes, membranes were incubated in Western-Lightning Plus-ECL (Perkin Elmer) and visualized using the Odyssey Fc imaging system (Li-Cor).

For detection of stably expressed proteins, 5E+06 cells were washed using PBS and processed as described above.

### Co-immunoprecipitation

pHT1 and/or pTat and/or empty pEF.Bos vector (see above), were transfected using Lipofectamine 3000 (Invitrogen) in 3.5E+06 293T cells. Transfection amounts were as follow: lane 1, 500 ng/mL pHT1 and 500ng mL pTat; lane 3, 500 ng/mL pHT1; lane 4, 500 ng/mL pTat; lanes 5–7: 500 ng/mL pTat and 250, 500 or 1,000 ng/mL pHT1; in all lanes, pEF-Bos qsp 1.5 μg/mL total DNA. After 48 hrs, the cells were washed using PBS, and lysed using RIPA buffer containing 150 mM KCl and a protease inhibitor mixture (Thermo Fisher Scientific). Lysates were precleared for 2 hrs at 4°C using protein G-sepharose beads (Life Technologyies, and the precleared lysates were incubated overnight at 4°C with 4 μg of the indicated primary Ab or control mouse IgG1 (MI10-102, Bethyl). Protein G-sepharose beads were added to the lysates and incubated for 2 hrs at 4°C, washed five times with RIPA buffer, resuspended in 2X Laemmli buffer (BioRad) supplemented with DTT and incubated for 10 min at 95°C. Supernatants were subjected to WB as described above.

### *In vitro* kinase assay

A total of 30 μg pHT1 or pTat was transfected in 4E+06 293T cells using Lipofectamine 2000 (Invitrogen). 48 hrs after transfection, cells were lysed with buffer A (20 mM HEPES-KOH pH7.8, 0.3 M KCl, 0.1% Nonidet P-40 and 0.2mM EDTA), and m:HT1 or m:Tat proteins were immunoprecipitated by using Myc-Trap A kit (Chromotek). Immunoprecipitation with mouse IgG-coupled protein G sepharose was used as a negative control. After washing three times with buffer A, beads were washed once with CTD kinase buffer (20 mM HEPES-NaOH, 7.5 mM MgOAc, 2%Glycerol, 0.1 M KOAc, 2 mM DTT). Beads were then incubated with 30 μL CTD kinase buffer containing 0.25 μg of purified recombinant GST-CTD proteins (Sigma) and 50 mM ATP for 60 min at 37°C. Kinase reactions were terminated by adding 30 mL 2x Laemmli sample buffer (BioRad), and heating the mixture for 5 min at 95°C. Supernatants were subjected WB, and phosphorylated GST-CTD proteins (P-CTD) were detected by anti-phospho CTD (Ser 2, ab5094, Abcam). Co-immunoprecipitated P-TEFb (CDK9) was also detected by WB. Band intensities of P-CTD and CDK9 were quantified by LiCor imaging system, and relative kinase activities were calculated as P-CTD band intensities normalized by CDK9 band intensities. Six replicate experiments were performed.

### RNA-IP

pHT1 and/or pTat and/or empty pEF.Bos vector (see above) and pU16TAR [3] (a generous gift from Dr. John Rossi at City of Hope) for TAR RNA expression from an RNAPIII promoter, were transfected in 4.0E+06 293T cells using Lipofectamine 2000 (Invitrogen). In Fig 2C, transfected amounts were 500 ng/mL pU16TAR and 1.5 μg/mL pEF-Bos, pHT1 or pTat. In Fig 2D, transfected amounts were 500 ng/mL pU16TAR, 1.0 μg/mL pTat and 0.5, 1.0 or 1.5 μg/mL pHT1, plus pEF-Bos qsp 3 μg/mL total DNA. The cells were lysed in buffer A on ice for 20 min. Cell lysates were centrifuged at 14,000 rpm for 5 min at 4°C, and supernatants were collected. Cell lysates were then precleared with protein G-Sepharose beads (Invitrogen) and divided into two aliquots. Each aliquot was incubated with 1 μg of normal-rabbit IgG or anti-Myc (ab9106, Abcam) Abs precoupled with Protein-A dynabeads (Life Techonologies), or anti-Flag (M2)-conjugated magnetic beads (Sigma) for 2–4 hrs at 4°C. Beads were washed five times with buffer A. RNA was then extracted with TRIzol (Invitrogen), followed by DNase I treatment (Turbo DNAfree kit, Ambion). Reverse transcription quantitative PCR (RT-qPCR) analyses were performed using Superscript III First Strand synthesis system (Invitrogen) with 1 μL random hexamers (Invitrogen) and 1 μL RNaseOUT (Invitrogen), and then sensiFAST Lo Rox kit (Bioline), to quantify TAR RNA enriched in the immunoprecipitates. The same sets of qPCR analyses using the samples without reverse transcription confirmed that the DNA contamination from transfected plasmid reporters was negligible. Sequences for specific primers for TAR RNA or 7SK snRNA are as follows: TAR, 5’-CTTACTCTGTTCTCAGCGACA-3’ (forward) and 5’-CAACCTTCTGTACCAGCTTACT-3’ (reverse), 7SK,5'-GAGGGCGATCTGGCTGCGACAT-3' (forward) and 5'-ACATGGAGCGGTGAGGGAGGAA-3' (reverse) [29]. Known concentrations of the pU16TAR plasmids were used as standards to determine the copy number of TAR RNA by qPCR analysis. Data are shown by relative TAR enrichment by calculating values obtained with anti-Myc- or anti-Flag-immunoprecipitations divided by values obtained with IgG controls.

### HEXIM1 RT-qPCR

A total of 2.0 μg DNA, including pHT1 and/or empty pEF.Bos vector (see above), was transfected using Lipofectamine 3000 (Invitrogen) in 8.0E+05 293T cells. After 48 hrs, the cells were washed using PBS, lysed using TRIzol (Invitrogen), and RNA was purified using DirectZol RNA kit (zymo research) followed by DNase I treatment (Turbo DNA free Ambion). 500 ng RNA was used for reverse transcription using Superscript III First Strand synthesis system (Invitrogen) with 1 μL random hexamers (Invitrogen) and 1 μL RNAseOUT (Invitrogen). Resulting cDNA was then used for HEXIM1 and GAPDH qPCR using sensiFAST Lo Rox kit (Bioline) and the following primers: 5’-CACCAGCGATGACGACTT-3’ (forward) and 5’-TCATGTTCTGCAGGCTCT-3’ (reverse) for HEXIM1, 5’-ACCACAGTCCATGCCATCAC-3’ (forward) and 5’-TCCACCACCCTGTTGCTGTA-3’ (reverse) for GAPDH. Each condition was tested in triplicate experiments, and results are shown as mean HEXIM1 mRNA count relative to GAPDH, normalized to the mean relative count in the control condition (pHT1 = 0 ng/mL).

### mRNA-seq

A total of 2.0 μg DNA (pHT1 or empty pEF.Bos vector, see above), was transfected using Lipofectamine 3000 (Invitrogen) in 8.0E+05 293T cells. After 48 hrs, transfected 293T cells and CycT1 KO 293T cells were washed using PBS, lysed using TRIzol (Invitrogen), and RNA was purified using DirectZol RNA kit (Zymo research) followed by DNase I treatment (Turbo DNA free Ambion). 500 ng RNA was used to prepare mRNA libraries using QuantSeq 3’ mRNA kit (Lexogen) for Illumina. High Sensitivity DNA kit (Agilent) was used for quality control of the libraries using a Bioanalyzer (Agilent). Libraries were sequenced using a HiSeq4000 (Illumina), and the reads were analyzed using the differential expression pipeline from BlueBee, using empty vector transfected 293T cells as a control for HT1-expressing 293T cells and for CycT1 KO 293T cells. Each condition was tested in triplicate experiments.

### HIV reactivation

5.0E+05 2D10 or D-HT cells were treated with PMA (10 nM), SAHA (5 μM) or JQ1 (5 μM) and 5.0E+05 J-Lat 9.2 or J-HT cells were treated with PMA (100 nM). After 24h, the cells were washed with PBS containing 2% FBS, then fixed in 2% paraformaldehyde. A BD LSRII FACS analyzer was then used to determine the percentage of GFP-expressing cells as a proxy for HIV reactivation. Each condition was tested in triplicate experiments.

### HIV infection

Wild type HIV-1 molecular clone pNL43 (given by Dr. Oliver Fackler, University of Heidelberg) was transfected in 293T cells using Polyplus transfection kit (Jetprime). 72 hrs after transfection, the supernatant from the 293T cell culture was filtered and used for infection of 1.0E+06 cells in the presence of 2 μg/mL polybrene. HIV infection was completed by spinoculation for 90min at 2,500 rpm. After 24 hrs, the cells were extensively washed in PBS and resuspended in 5 mL fresh medium. Day 0 supernatant (1mL) was collected before the cells were cultured further. The cells were passaged on Days 2 and 4, and 1mL supernatant was collected before each passage. HIV replication was measured by detection of Gag p24 in the supernatants by ELISA using HIV-1 p24 antigen capture assay (ABL). Each condition was tested in triplicate experiments.

### HIV provirus measurement

Replication-defective HIV Env-pseudotyped HIV-1 was produced as described above by co-transfecting pNL43ΔenvLuc and pHXB2-env (given by Dr. Emilie Battivelli, Buck Institute for Research on Aging) in 293T cells and collecting supernatants. The same amount of virus-containing supernatant was mixed with cells (1.0E+06) in the presence of 2 μg/mL polybrene. Cells were further incubated overnight, washed once, and resuspended with fresh media. 24 hours later, cells were harvested and genomic DNA was isolated using QIAamp DNA Blood Mini kit (QIAGEN). 100ng of DNA was subjected to qPCR analysis with specific primers: 5’-ACCCTGAACTAGCAGACCAACT-3’ (forward) and 5’-ACACTAGGCAAAGGTGGCTT-3’ (reverse) for HIV (H9 and H10 in [39]) and 5’-TCAAGTGGGGCGATGCTGGC-3’ (forward) and 5’- TGGGGGCATCAGCAGAGGGG-3’ for genomic GAPDH. Each condition was tested in triplicate experiments and results are shown as mean HIV DNA count relative to GAPDH.

## Supporting information

S1 Fig Amino acid sequences of the chimeras included in the study. They all include Myc-epitope tag (EQKLISEEDL). HT1(PNND) is a PYNT mutant of HT1. GGGS are linker sequences inserted between Hex(150–220) and Tat(1–48) domains of HT1. PID is the P-TEFb interaction domain from Brd4. 1TH is a swapped-domain mutant of HT1 where Hex(150–220) and Tat(1–48) domains have been inverted.
(TIFF)

S2 Fig **A. Activity of various chimeras on Tat-induced LTR-driven Luc expression.** As in Fig 1B, pLTR-Luc was co-transfected with pTat and a pHT plasmid for expression of the indicated chimera in 293T cells (pHT : pTat ratio = 1 : 2). Luc activity was plotted as % activity relative to control (EV = empty vector used instead of pHT). Error bars in the graph represent standard deviation from triplicate experiments. Lower panel: expression levels of m:HT and f:Tat were controlled by WB using anti-Myc and anti-Flag Abs. Housekeeping protein β-actin was used as loading control. B. **Transient expression of HT1 inhibits Tat-induced LTR-driven Luc expression in NH1 cells, which stably carry an LTR-Luc reporter gene.** pTat and a pHT plasmid for expression of the indicated chimera were co-transfected in NH1 cells (pHT : pTat ratio = 1 : 2). Luc activity was plotted as % activity relative to control (EV = empty vector used instead of pHT). Error bars in the graph represent standard deviation from triplicate experiments.
(TIFF)

S3 Fig **A. HT1 and HT2, but not HT3 binds to TAR.** m:HT1, m:HT2, or m:HT3 (or empty vector, EV, as a control) was transiently co-expressed with TAR RNA-expressing pU16TAR in 293T cells. Cell lysates were used for IP using anti-Myc and submitted to RT-qPCR using TAR-specific primers. Relative TAR enrichment was calculated as in Fig 2C. **B. HT1 binds to 7SK snRNA.** m:HT1 (or empty vector, EV, as a control) was transiently expressed in 293T cells. Cell lysates were used for IP using anti-Myc Ab or control IgG. RNA was purified from the immunoprecipitates and submitted to RT-qPCR using 7SK-specific primers. Relative 7SK snRNA enrichment was calculated by qPCR, and normalized to EV. Error bars represent standard deviation from triplicate qPCR assays.
(TIFF)
