# Supplementary figures and images for: HEXIM1-Tat chimera inhibits HIV-1 replication
Source: PLoS Pathog. 2018 Nov 5;14(11):e1007402. doi: 10.1371/journal.ppat.1007402 (PMC6245832; doi:10.1371/journal.ppat.1007402)

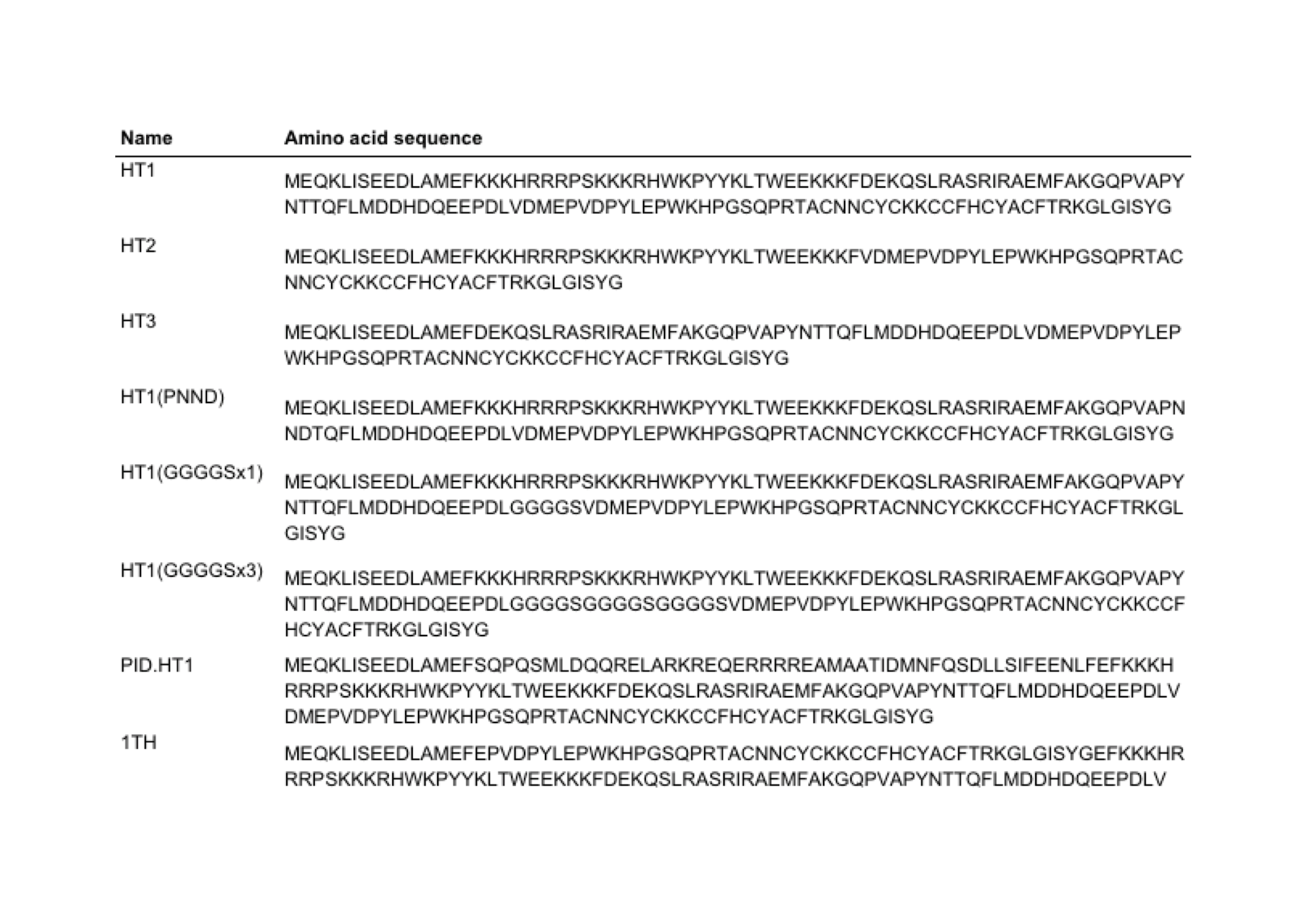

Supplement: S1 Fig — They all include Myc-epitope tag (EQKLISEEDL). HT1(PNND) is a PYNT mutant of HT1. GGGS are linker sequences inserted between Hex(150–220) and Tat(1–48) domains of HT1. PID is the P-TEFb interaction domain from Brd4. 1TH is a swapped-domain mutant of HT1 where Hex(150–220) and Tat(1–48) domains have been inverted. (TIFF) [file ppat.1007402.s001.tiff]

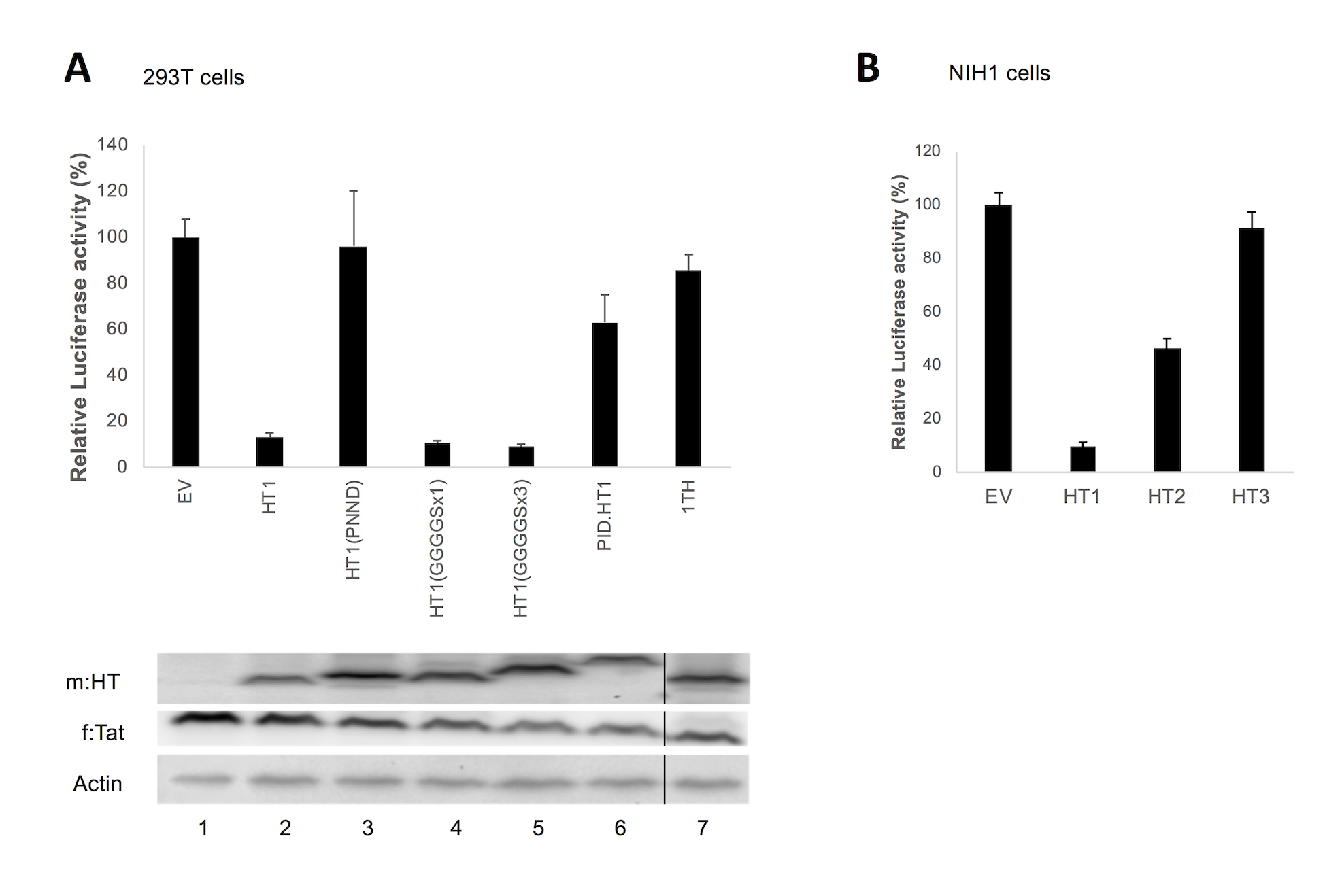

Supplement: S2 Fig — A. Activity of various chimeras on Tat-induced LTR-driven Luc expression. As in Fig 1B, pLTR-Luc was co-transfected with pTat and a pHT plasmid for expression of the indicated chimera in 293T cells (pHT : pTat ratio = 1 : 2). Luc activity was plotted as % activity relative to control (EV = empty vector used instead of pHT). Error bars in the graph represent standard deviation from triplicate experiments. Lower panel: expression levels of m:HT and f:Tat were controlled by WB using anti-Myc and anti-Flag Abs. Housekeeping protein β-actin was used as loading control. B. Transient expression of HT1 inhibits Tat-induced LTR-driven Luc expression in NH1 cells, which stably carry an LTR-Luc reporter gene. pTat and a pHT plasmid for expression of the indicated chimera were co-transfected in NH1 cells (pHT : pTat ratio = 1 : 2). Luc activity was plotted as % activity relative to control (EV = empty vector used instead of pHT). Error bars in the graph represent standard deviation from triplicate experiments. (TIFF) [file ppat.1007402.s002.tiff]

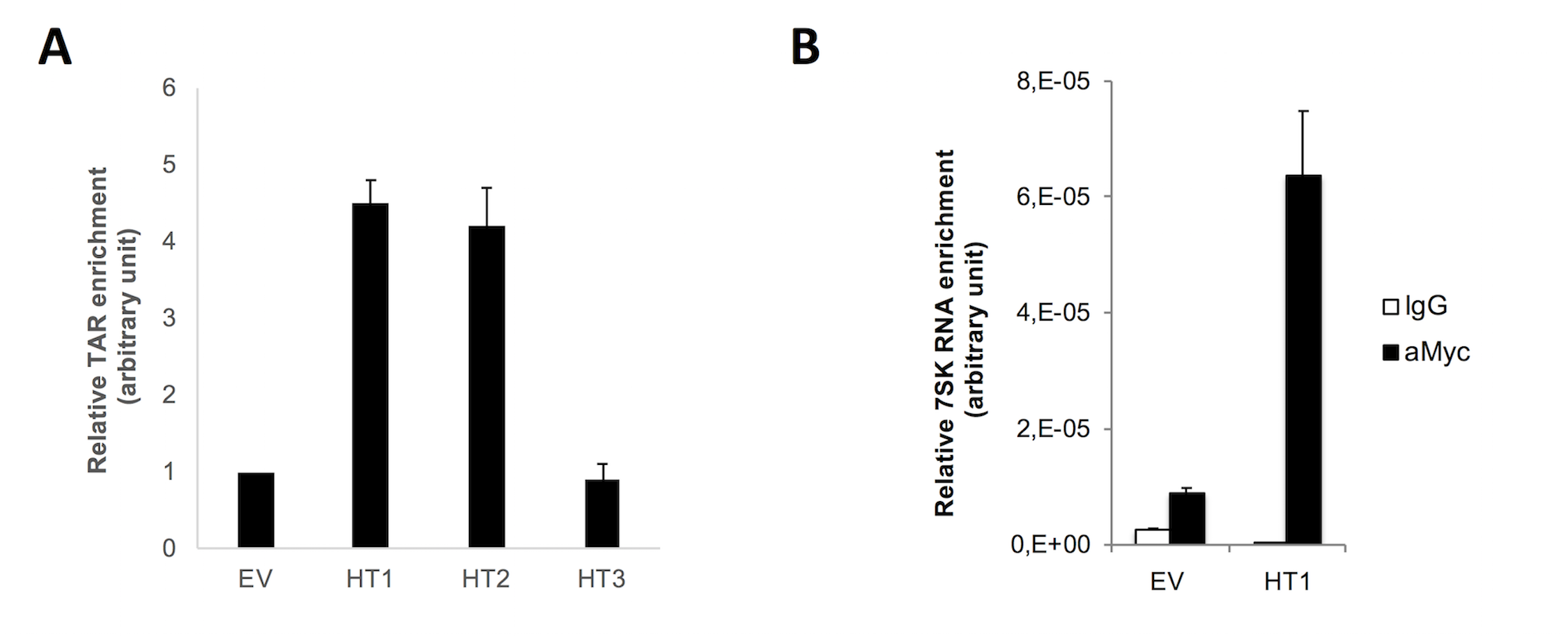

Supplement: S3 Fig — A. HT1 and HT2, but not HT3 binds to TAR. m:HT1, m:HT2, or m:HT3 (or empty vector, EV, as a control) was transiently co-expressed with TAR RNA-expressing pU16TAR in 293T cells. Cell lysates were used for IP using anti-Myc and submitted to RT-qPCR using TAR-specific primers. Relative TAR enrichment was calculated as in Fig 2C. B. HT1 binds to 7SK snRNA. m:HT1 (or empty vector, EV, as a control) was transiently expressed in 293T cells. Cell lysates were used for IP using anti-Myc Ab or control IgG. RNA was purified from the immunoprecipitates and submitted to RT-qPCR using 7SK-specific primers. Relative 7SK snRNA enrichment was calculated by qPCR, and normalized to EV. Error bars represent standard deviation from triplicate qPCR assays. (TIFF) [file ppat.1007402.s003.tiff]
